# Genetically confirmed limb-girdle muscular dystrophy type 2B with DYSF mutation using gene panel sequencing: A case report

**DOI:** 10.1097/MD.0000000000020810

**Published:** 2020-07-10

**Authors:** Sook Joung Lee, Eunseok Choi, Soyoung Shin, Joonhong Park

**Affiliations:** a Department of Physical Medicine and Rehabilitation; b Department of Laboratory Medicine, College of Medicine, The Catholic University of Korea, Seoul, Republic of Korea.

**Keywords:** *DYSF* mutation, gene panel sequencing, genetic diagnosis, limb-girdle muscular dystrophy

## Abstract

**Rationale:**

The limb-girdle muscular dystrophies (LGMDs) are a heterogeneous group of disorders characterized by progressive proximal muscle weakness and have more than 30 different subtypes linked to specific gene loci, which manifest as highly overlapping and heterogeneous phenotypes.

**Patient concerns:**

A 59-year-old male presented for evaluation of progressive muscle weakness since his late twenties. When he was 38 years old, he had muscle weakness in the upper extremities and had a waddling gait, hyper lordosis of lower back, and anterior pelvic tilt. His gait disturbance and muscle weakness slowly progressed. When he was 55 years old, he could not walk at all and had to use a wheelchair for ambulation.

**Diagnosis:**

Next-generation sequencing using a custom target capture-based gene panel including specific genes responsible for muscular dystrophy was performed. As a result, the proband was genetically diagnosed as LGMD type 2B, carrying 2 compound heterozygous mutations (NM_003494.3:c.1663C>T, p.Arg555Trp; rs377735262 and NM_003494.3:c.2997G>T, p.Trp999Cys; rs28937581) of the *DYSF* gene.

**Interventions:**

Physical and occupational therapy were prescribed properly for the first time Bracing and assistive devices were adapted specifically to the patient's deficiencies to preserve mobility and function and prevent contractures.

**Outcomes:**

The patient with LGMD has periodic assessments of physical and occupational therapy for the prevention and management of comorbidities. However, in the 3 years after the gene panel sequencing diagnoses, his weakness was slowly progress and the patient still could not walk.

**Lessons:**

Gene panel sequencing allows for the correct recognition of different LGMD subtypes, improving timely treatment, management, and enrolment of molecularly diagnosed individuals in clinical trials.

## 1 Introduction

Limb-girdle muscular dystrophies (LGMD) are a heterogeneous group that causes progressive muscle weakness and wasting of the muscles in the shoulder and pelvic girdle. Although each of the individual LGMD subtypes is relatively rare, a recent meta-analysis estimated LGMDs prevalence as 1.63 per 100 000 individuals, ranging from 0.37 in China to 1.55 per 100 000 individuals in Japan and is one of the most common muscular dystrophies .^[1]^ LGMDs are classified according to the inheritance mode into LGMD1/LGMD-D, with an autosomal dominant manner, and LGMD2/LGMD-R, with an autosomal recessive manner. Among these groups, each specific subtype is designated by a letter or number given in the chronological order of locus mapping in the novel nomenclature systems .^[2]^ The substantial variability and overlap among each LGMD subtype in the age of onset, affected muscle groups, and severity, and the fact that cost of molecular testing was previously unaffordable for patients, make definitive diagnosis highly elusive .^[3]^ Considering the growing number of subtypes and their clinical overlap, the lack of specific tests to identify protein defects for most recently described subtypes, and the costs and laboriousness of conventional Sanger sequencing diagnosis for genetically heterogeneous disorders, target or unbiased next-generation sequencing (NGS) is becoming widely used as both confirmatory and candidate approaches for LGMD diagnosis^[4–6]^

Here, we report a Korean case of familial LGMD type 2B caused by a compound missense mutation of the *DYSF* gene, using a gene panel sequencing approach to interrogate the genome of the patient. To the best of our knowledge, this is the first report of Korean LGMD type 2B with p.Arg555Trp of the *DYSF* gene, one of the compound missense mutations.

## 2 Case presentation

**Figure 1:**
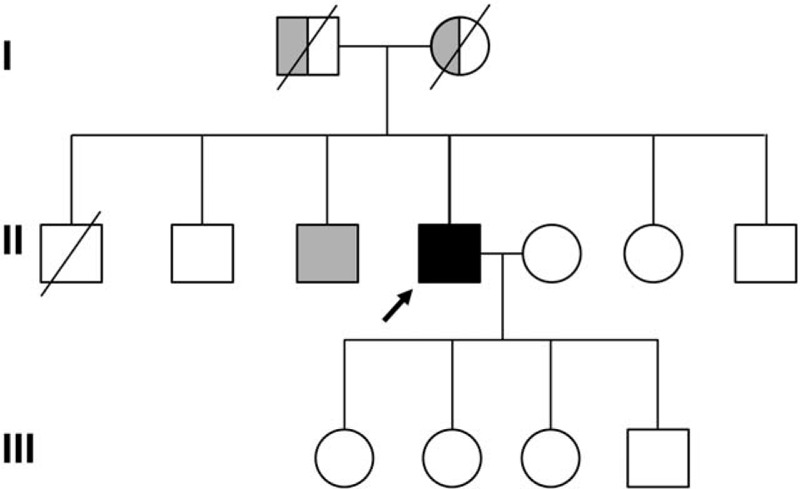
Pedigree analysis of the proband (arrow) with compound heterozygous mutation in *DYSF* gene. Gray symbol, clinically suspected but the result of genetic study was not available.

**Figure 2:**
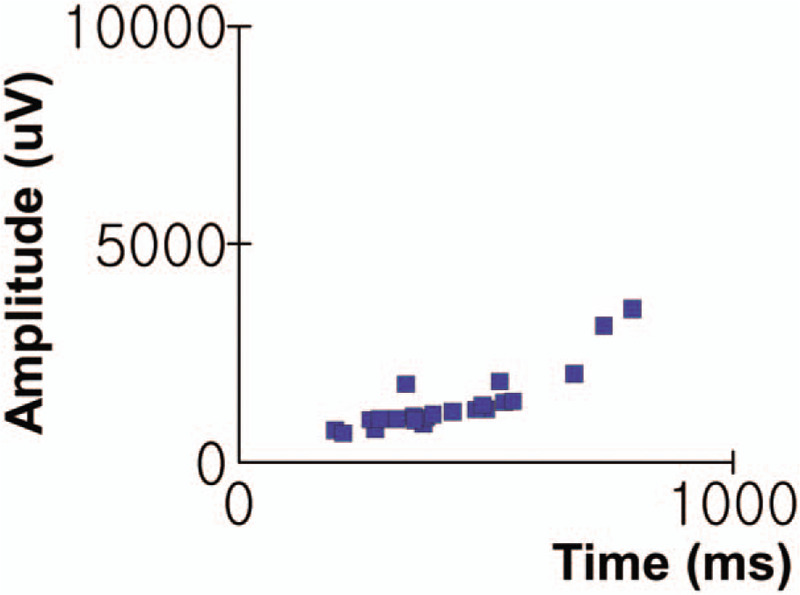
Electromyography in the proband. Quantitative electromyography showed myopathic pattern in left vastus medialis muscle.

A 59-year-old male (Fig. 1, individual II-4) presented for an evaluation of progressive muscle weakness since his late twenties at the outpatient department of the rehabilitation center, Daejeon St. Mary's Hospital (Daejeon, Republic of Korea). He was born healthy at full-term without problems. He was healthy during childhood and early adulthood and fulfilled mandatory national military training. In his late twenties, he fell down easily and had difficulty climbing stairs. He had progressive muscle weakness especially in the pelvic girdle and proximal leg. However, he described that the motor of the upper extremity was preserved as normal at that time. At this age, he was presumably diagnosed with some type of myopathy from electrodiagnostic study and laboratory study at the age of 28 years old. When he was 38 years old, he felt muscle weakness in the upper extremities and had waddling gait, hyper lordosis of lower back, and anterior pelvic tilt. His gait disturbance and muscle weakness slowly progressed. When he was 55 years old, he could not walk at all and had to use a wheelchair for ambulation. His family history shows genetic background of autosomal recessive inheritance on his myopathic condition. Among the proband's siblings, his 3rd elder brother (Fig. 1, individual II-3) had a similar clinical manifestation to the proband and was diagnosed as progressive muscular dystrophy by electrodiagnostic study and muscle biopsy in his twenties. The proband was married to a normal healthy woman and had healthy sons and daughters showing no myopathic symptoms. In the physical examination, muscle power was decreased in both the upper and lower extremities, and proximal weakness was predominant (shoulder and hip girdle: poor; elbow, wrist, hand, and ankle: fair to poor). The deep tendon reflex was decreased, and no pathologic reflex was checked. The patient had multiple joint contractures in both hips, shoulders, and ankle joints. He also showed muscle atrophy in all extremities, which was especially severe in the pelvic girdle and shoulder girdle muscles. In the laboratory findings, the serum creatine kinase (CK) level was elevated in 2042 IU/L. His cardiac markers (NT-proBNP, CK-MB, Troponin T) were within the normal range, and the echocardiogram showed normal function. His pulmonary function test was within the normal limit, and his forced vital capacity was 4.70 liters (95% of the estimated level). The electrodiagnostic study revealed myopathy, normal values in the motor and sensory nerve conduction study, and short-amplitude muscle unit action potentials with early recruitment in needle electromyography. Quantitative electromyography also showed a myopathic pattern (Fig. 2). Muscle biopsy could not be conducted. The patient with LGMD has periodic assessments of physical and occupational therapy for the prevention and management of comorbidities. However, in the 3 years after the gene panel sequencing diagnoses, his weakness was slowly progress and the patient still could not walk.

## 3 Genetic analyses

**Figure 3:**
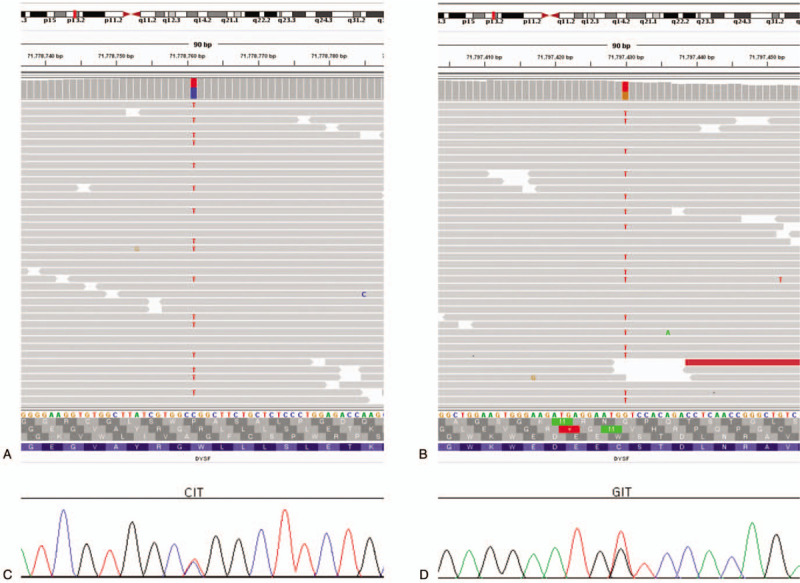
Gene panel sequencing and Sanger sequencing in the proband. A and B. Gene panel sequencing revealed 2 compound heterozygous mutations in the *DYSF* gene: (A) c.1663C>T; p.Arg555Trp and (B) c.2997G>T; p.Trp999Cys. C and D. Sanger sequencing confirmed the presence of each of the 2 alterations in heterozygous state: (C) c.1663C>T and (D) c.2997G>T of the *DYSF* gene.

To identify the underlying genetic cause of his progressive muscle weakness, a blood sample of the proband only was obtained, and NGS using a custom target capture-based gene panel including specific genes responsible for muscular dystrophy was performed (Table 1 ). The study protocol was approved by the Institutional Review Board of the Catholic University of Korea. Study subject provided written informed consent for clinical and molecular analysis. Briefly, capture-based target enrichment was performed using custom probes and the SureSelect^QXT^ Target Enrichment Kit (Agilent Technologies, Santa Clara, CA). Massively parallel sequencing was performed on the Illumina HiSeq 2000 platform (Illumina Inc., San Diego, CA). Raw sequencing data pre-processing and initial variant calling were performed according to the GATK Best Practices workflows for germline short variant discovery (https://software.broadinstitute.org/gatk/). Common variants with allele frequency > 0.01 from the large-scale re-sequencing studies (TOPMed, Exome Aggregation Consortium, and 1000 Genomes Project) were excluded. The remaining single nucleotide variants (SNVs) and small insertion and deletions (Indels) with Phred quality score > 20 and coverage depth > 100 × were estimated to be pathogenic by referring to the neuromuscular diseases-associated variants in disease-related databases (ClinVar, HGMD, and OMIM). As a result, compound heterozygous C-to-T base substitution at position 1663 in exon 19 of the *DYSF* gene, leading to an amino acid change from arginine to tryptophan at position 555 (NM_003494.3:c.1663C> T, p.Arg555Trp; rs377735262), along with a heterozygous G-to-T base change at position 2997 in exon 28 of the *DYSF* gene, causing a codon change from tryptophan to cysteine at position 999 (NM_003494.3:c.2997G>T, p.Trp999Cys; rs28937581), were identified as candidate causative mutations (Fig. 3A and 3B). These 2 suspected mutations in *DYSF* identified in the proband were subsequently confirmed as compound heterozygous by Sanger sequencing (Fig. 3C and 3D). According to the autosomal recessive manner and proximal muscle weakness in lower limbs, the proband was genetically diagnosed as LGMD type 2B, carrying 2 compound heterozygous mutations of the *DYSF* gene (Table 2).

**Table 1:**
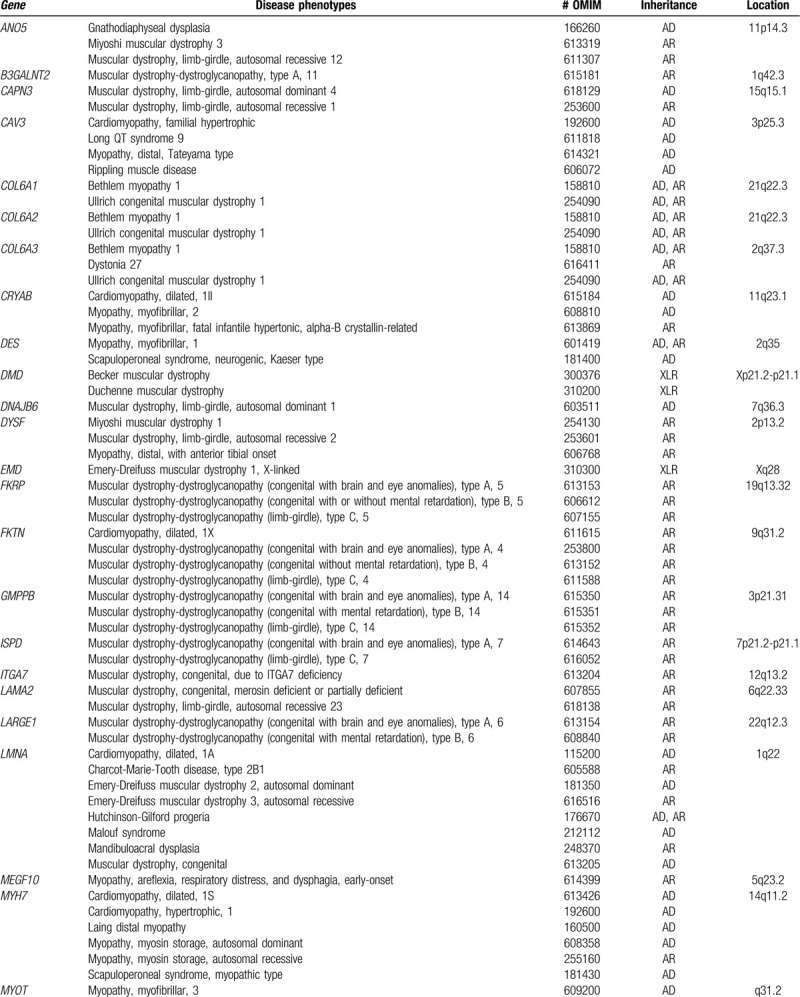
Gene panel list for limb-girdle muscular dystrophies and inherited muscular dystrophies.

**Table 1 (Continued):**
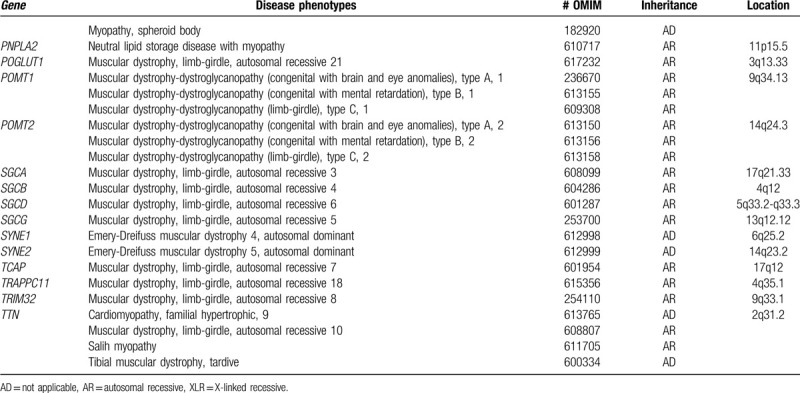
Gene panel list for limb-girdle muscular dystrophies and inherited muscular dystrophies.

**Table 2:**
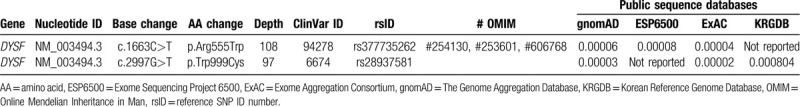
Details for compound heterozygous mutations of DYSF in a patient with Limb-girdle muscular dystrophy, type 2B.

## 4 Discussion

Dysferlinopathies (LGMD type 2B and Miyoshi myopathy) include autosomal-recessive muscle diseases caused by pathogenic variants in the dysferlin gene (*DYSF*), characterized by a selective and progressive involvement of the proximal and/or distal muscles of the limb girdles.^[7]^ The age at onset of muscle weakness varies widely, but usually occurs in the teenage years or early adulthood. The serum CK level is highly elevated from the early asymptomatic stage of the disease and is a hallmark of dysferlinopathy.^[8]^ The causative gene, *DYSF* encodes a protein called “dysferlin,” the muscle-specific member of a class of homologous proteins termed “ferlins.” Dysferlin has been recognized as having a crucial role in the active process of repairing muscle membrane lesions and acts as a key Ca^2+^ ion sensor that triggers vesicle and membrane fusion by binding its C2-domain to phospholipids in a Ca^2+^-dependent manner.^[9]^*DYSF*, *CAPN3*, and *COL6A1* have the highest number of pathogenic variants, indicating more allelic heterogeneity in these genes compared to others in different LGMD-associated genes.^[10]^

We reported the utility of gene panel sequencing for genetic diagnosis with regard to a Korean LGMD type 2B who was initially suspected to have unknown myopathy. The patient in our study represents a challenge for diagnosis because he had previously been investigated using electrodiagnostic study and remained without a diagnosis for his conditions. Moreover, no large exonic deletion or duplication of the *DMD* gene was detected by gene dosage study using multiplex ligation-dependent probe amplification at initial genetic work-up. Duchenne/Becker muscular dystrophy patients with milder presentations often mimic limb-girdle phenotypes, and common genetic neuromuscular disorders that overlap with LGMDs are routinely missed when using NGS technologies owing to DNA expansions/deletions.^[11]^ Actually, it is difficult for clinicians to provide an accurate clinical diagnosis based on limb-girdle phenotypes due to overlapping phenotypes and complex presentations. Usually, the severity of the different LGMD subtypes varies, with most of the dominant forms having later-onset ages and milder-clinical presentations than the recessive subtypes.^[12]^ Muscle biopsy was not required after genetic diagnosis using the NGS. Although the patients had been received consistent rehabilitation therapy for weakness and functional improvement, his symptoms were not improved.

In this case, recurrent p.Arg555Trp^[13–16]^ and p.Trp999Cys^[17–21]^ of the *DYSF* mutations were identified by gene panel sequencing and have been reported in different ethnic population groups across the country. According to a review of the literature on genetic analysis for Korean LGMD,^[20,22–25]^ the first reported case of Korean Miyoshi myopathy with p.Glu389Gln of the *DYSF* was genetically confirmed in 2004, and several studies using targeted NGS with an LGMD-related gene panel for inherited muscular dystrophies in the Korean population have been reported.^[20,23]^ In another study based on Sanger sequencing, 2 common mutations (c.663+1G>C and p.Gln832∗) accounted for approximately one-third of the *DYSF* mutations in Korean patients with dysferlinopathy and Korean *DYSF* mutations appeared to cluster in the N-terminal region.^[24]^ To date, p.Trp999Cys of the *DYSF* has been reported recurrently in dysferlinopathy,^[20,22–24]^ but not p.Arg555Trp of the *DYSF* gene. Additionally, the former (rs28937581) was present as 0.000804, but the latter (rs377735262) was not reported in 622 ethnicity-matched control subjects of Korean descent in the Korean Reference Genome Database (http://coda.nih.go.kr/coda/KRGDB/index.jsp). The p.Arg555Trp mutation lies in a polypeptide stretch between the C2C domain and the Dysf domain of the dysferlin protein, a region that may have low functional relevance. It is conceivable that many other dysferlin missense mutant proteins may retain their functional activity when salvaged from degradation. Inhibition of the proteasome by lactacystin or Velcade increases the levels of p.Arg555Trp mutated dysferlin. This salvaged protein is functional because it restores plasma membrane resealing in patient-derived myoblasts and reverses their deficit in myotube formation.^[26]^ Likewise, proteasome inhibitor (MG-132) improved the expression level and biological function of missense mutated p.Trp999Cys dysferlin and proteasomal inhibition was not effective in increasing frameshift or nonsense mutated dysferlin protein levels.^[27]^ Thus, the possibility that inhibition of the degradation pathway of missense mutated dysferlin could be used as a therapeutic strategy for patients harboring certain dysferlin missense mutations.

On the other hand, genetic studies using NGS in large clinically characterized patient cohorts have improved knowledge of the gene-variant spectrum, mutational hotspots, genetic etiologies, and relative prevalence of different LGMD subtypes, allowing timely management, participation of definitively diagnosed individuals in ongoing clinical trials through disease-specific registries.^[10,28,29]^ Along with a review of the clinical phenotype, follow-up investigations of biopsy specimens, serum enzyme assays, and/or MRI supporting the genetic diagnosis identified by use of NGS, transferring NGS to clinical practice is crucial not only to facilitate diagnosis but also to improve optimal health outcomes of patients by targeted health surveillance.^[30,31]^

## 5 Conclusion

To our knowledge, this is the first report of Korean LGMD type 2B carrying p.Arg555Trp of the *DYSF* gene, along with p.Trp999Cys of the *DYSF* gene as compound heterozygous state. Gene panel sequencing is recommended as a common test for patients with LGMD because LGMD has more than 30 different subtypes linked to specific gene loci, which manifest in highly overlapping and heterogeneous phenotypes. This high-throughput sequencing technology allows for the correct recognition of different LGMD subtypes, improving timely treatment, management, and enrolment of molecularly diagnosed individuals in clinical trials.

## Acknowledgments

This work was supported by the National Research Foundation of Korea (NRF) grant funded by the Korea government (MSIT) (2020R1F1A1077316).

## Author contributions

SJL and EC analyzed the data and drafted the manuscript, SS participated in the critical revision of the manuscript, and JP performed the experiments and drafted the manuscript, and finalized the manuscript.

## References

[1] Mah JK Korngut L Fiest KM A systematic review and meta-analysis on the epidemiology of the muscular dystrophies. Can J Neurol Sci 2016;43:163–77. 26786644 10.1017/cjn.2015.311

[2] Straub V Murphy A Udd B 229th ENMC international workshop: limb girdle muscular dystrophies - Nomenclature and reformed classification Naarden, the Netherlands, 17-19 March 2017. Neuromuscul Disord 2018;28:702–10. 30055862 10.1016/j.nmd.2018.05.007

[3] Simeoni S Russo V Gigli GL Facioscapulohumeral muscular dystrophy and limb-girdle muscular dystrophy: “double trouble” overlapping syndrome? J Neurol Sci 2015;348:292–3. 25528007 10.1016/j.jns.2014.12.009

[4] Bartoli M Desvignes JP Nicolas L Exome sequencing as a second-tier diagnostic approach for clinically suspected dysferlinopathy patients. Muscle Nerve 2014;50:1007–10. 25046369 10.1002/mus.24344

[5] Monies D Alhindi HN Almuhaizea MA A first-line diagnostic assay for limb-girdle muscular dystrophy and other myopathies. Hum Genomics 2016;10:32–8. 27671536 10.1186/s40246-016-0089-8 PMC5037890

[6] Ozyilmaz B Kirbiyik O Ozdemir TR Impact of next-generation sequencing panels in the evaluation of limb-girdle muscular dystrophies. Ann Hum Genet 2019;83:331–47. 31066050 10.1111/ahg.12319

[7] Liu J Aoki M Illa I Dysferlin, a novel skeletal muscle gene, is mutated in Miyoshi myopathy and limb girdle muscular dystrophy. Nat Genet 1998;20:31–6. 9731526 10.1038/1682

[8] Fanin M Angelini C Progress and challenges in diagnosis of dysferlinopathy. Muscle Nerve 2016;54:821–35. 27501525 10.1002/mus.25367

[9] Bansal D Miyake K Vogel SS Defective membrane repair in dysferlin-deficient muscular dystrophy. Nature 2003;423:168–72. 12736685 10.1038/nature01573

[10] Nallamilli BRR Chakravorty S Kesari A Genetic landscape and novel disease mechanisms from a large LGMD cohort of 4656 patients. Ann Clin Transl Neurol 2018;5:1574–87. 30564623 10.1002/acn3.649 PMC6292381

[11] Murphy AP Straub V The classification, natural history and treatment of the limb girdle muscular dystrophies. J Neuromuscul Dis 2015;2:S7–19. 10.3233/JND-150105 PMC5271430 27858764

[12] Moore SA Shilling CJ Westra S Limb-girdle muscular dystrophy in the United States. J Neuropathol Exp Neurol 2006;65:995–1003. 17021404 10.1097/01.jnen.0000235854.77716.6c

[13] Nguyen K Bassez G Bernard R Dysferlin mutations in LGMD2B, Miyoshi myopathy, and atypical dysferlinopathies. Hum Mutat 2005;26:165–75. 10.1002/humu.9355 16010686

[14] Krahn M Beroud C Labelle V Analysis of the DYSF mutational spectrum in a large cohort of patients. Hum Mutat 2009;30:E345–75. 18853459 10.1002/humu.20910

[15] Xi J Blandin G Lu J Clinical heterogeneity and a high proportion of novel mutations in a Chinese cohort of patients with dysferlinopathy. Neurol India 2014;62:635–9. 25591676 10.4103/0028-3886.149386

[16] Izumi R Niihori T Takahashi T Genetic profile for suspected dysferlinopathy identified by targeted next-generation sequencing. Neurol Genet 2015;1:e36–47. 27066573 10.1212/NXG.0000000000000036 PMC4811388

[17] Takahashi T Aoki M Tateyama M Dysferlin mutations in Japanese Miyoshi myopathy: relationship to phenotype. Neurology 2003;60:1799–804. 12796534 10.1212/01.wnl.0000068333.43005.12

[18] Takahashi T Aoki M Suzuki N Clinical features and a mutation with late onset of limb girdle muscular dystrophy 2B. J Neurol Neurosurg Psychiatry 2013;84:433–40. 23243261 10.1136/jnnp-2011-301339 PMC3595148

[19] Jin SQ Yu M Zhang W Dysferlin gene mutation spectrum in a large cohort of Chinese patients with dysferlinopathy. Chin Med J (Engl) 2016;129:2287–93. 27647186 10.4103/0366-6999.190671 PMC5040013

[20] Park HJ Jang H Kim JH Discovery of pathogenic variants in a large Korean cohort of inherited muscular disorders. Clin Genet 2017;91:403–10. 27363342 10.1111/cge.12826

[21] Dominov JA Uyan O McKenna-Yasek D Correction of pseudoexon splicing caused by a novel intronic dysferlin mutation. Ann Clin Transl Neurol 2019;6:642–54. 31019989 10.1002/acn3.738 PMC6469257

[22] Cho HJ Sung DH Kim EJ Clinical and genetic analysis of Korean patients with Miyoshi myopathy: identification of three novel mutations in the DYSF gene. J Korean Med Sci 2006;21:724–7. 16891820 10.3346/jkms.2006.21.4.724 PMC2729898

[23] Shin HY Jang H Han JH Targeted next-generation sequencing for the genetic diagnosis of dysferlinopathy. Neuromuscul Disord 2015;25:502–10. 25868377 10.1016/j.nmd.2015.03.006

[24] Park YE Kim HS Lee CH Two common mutations (p.Gln832X and c663+1G>C) account for about a third of the DYSF mutations in Korean patients with dysferlinopathy. Neuromuscul Disord 2012;22:505–10. 22297152 10.1016/j.nmd.2011.12.007

[25] Oh SH Kim TS Choi YC Identification of a dysferlin gene mutation in a Korean case with Miyoshi myopathy. Yonsei Med J 2004;45:927–30. 15515206 10.3349/ymj.2004.45.5.927

[26] Azakir BA Di Fulvio S Kinter J Proteasomal inhibition restores biological function of mis-sense mutated dysferlin in patient-derived muscle cells. J Biol Chem 2012;287:10344–54. 22318734 10.1074/jbc.M111.329078 PMC3323038

[27] Matsuda C Kiyosue K Nishino I Dysferlinopathy fibroblasts are defective in plasma membrane repair. PLoS Curr 2015;7:1–25. 10.1371/currents.md.5865add2d766f39a0e0411d38a7ba09c PMC4639325 26579332

[28] Ten Dam L Frankhuizen WS Linssen W Autosomal recessive limb-girdle and Miyoshi muscular dystrophies in the Netherlands: the clinical and molecular spectrum of 244 patients. Clin Genet 2019;96:126–33. 30919934 10.1111/cge.13544

[29] Winckler PB da Silva AMS Coimbra-Neto AR Clinicogenetic lessons from 370 patients with autosomal recessive limb-girdle muscular dystrophy. Clin Genet 2019;96:341–53. 31268554 10.1111/cge.13597

[30] Ghaoui R Cooper ST Lek M Use of whole-exome sequencing for diagnosis of limb-girdle muscular dystrophy: outcomes and lessons learned. JAMA Neurol 2015;72:1424–32. 26436962 10.1001/jamaneurol.2015.2274

[31] Reddy HM Cho KA Lek M The sensitivity of exome sequencing in identifying pathogenic mutations for LGMD in the United States. J Hum Genet 2017;62:243–52. 27708273 10.1038/jhg.2016.116 PMC5266644

